# Quality Improvement Project – Producing an Information Poster to Improve Easily Accessible Practical Information to Junior Doctors Whilst On-Call at Fieldhead Hospital

**DOI:** 10.1192/bjo.2024.452

**Published:** 2024-08-01

**Authors:** Harriet Winder-Rhodes, Amelia Thompson, Jamshid Nazari

**Affiliations:** South West Yorkshire Partnership NHS Foundation Trust, Wakefield, United Kingdom

## Abstract

**Aims:**

To create an information poster for the doctors’ on-call room and doctors’ office at Fieldhead Hospital (a Psychiatric Inpatient Hospital in Wakefield) to improve readily available practical information to doctors whilst on-call.

**Background** – Psychiatry on-call shifts can feel daunting, especially if this is the clinician's first (and perhaps only) exposure working as a doctor within this specialty. Psychiatric hospitals are not equipped to deal with physically unwell patients which can be challenging especially as the only junior doctor on-call out of hours. Although there is a comprehensive induction programme, doctors in training raised concerns that there is insufficient, readily available practical information whilst on-call.

**Methods:**

Surveys were sent out to doctors in training to ascertain their initial viewpoints about producing a poster and which information they feel should be included. Doctors included were foundation years, GP and core trainees on their psychiatry placement in the South West Yorkshire Partnership NHS Foundation Trust. Both qualitative (free text responses) and quantitative information (yes/no responses) were obtained via SurveyMonkey. An initial draft poster was produced and sent out to all doctors in training as well as the project lead and clinical lead. The poster was amended accordingly. The posters were printed and displayed in the on-call rooms and doctors’ office.

**Results:**

Four respondents responded to our initial pre-poster survey. They were highly receptive to the suggestion that this information would be in poster format to provide easily accessible information to help whilst on-call. Key topics identified for the poster included navigating logistical issues and information on-site, clerking new admissions and the relevant investigations required, important telephone numbers, personal safety and where and how to access relevant information and guidelines.

Feedback regarding the initial draft poster survey and the included information was also positive. Seven respondents replied and overall, they felt that the poster provided the relevant information. The project supervisor and clinical lead also provided constructive feedback and identified that locating risk assessments and discussing with a consultant when a patient is recalled to hospital on a CTO should also be included. The initial draft poster was amended following this feedback.

**Conclusion:**

In conclusion, we found that there was an unmet need for easy to access logistical information regarding on-call work. The on-call poster provided the necessary information in a succinct and clear manner which the trainees benefited from.

